# Dietary Yeast Cell Wall Improves Growth Performance and Prevents of Diarrhea of Weaned Pigs by Enhancing Gut Health and Anti-Inflammatory Immune Responses

**DOI:** 10.3390/ani11082269

**Published:** 2021-07-31

**Authors:** Jeong Jae Lee, Hyunjin Kyoung, Jin Ho Cho, Jeehwan Choe, Younghoon Kim, Yanhong Liu, Joowon Kang, Hanbae Lee, Hyeun Bum Kim, Minho Song

**Affiliations:** 1 Division of Animal and Dairy Science, Chungnam National University, Daejeon 34134, Korea; leejeongjae@gmail.com (J.J.L.); kyounghyunjin@gmail.com (H.K.); kangkr99@naver.com (J.K.); 2 Division of Food and Animal Science, Chungbuk National University, Cheongju 28644, Korea; jinhcho@cbnu.ac.kr; 3 Department of Beef Science, Korea National College of Agriculture and Fisheries, Jeonju 54874, Korea; choejhw@gmail.com; 4 Department of Agricultural Biotechnology and Research Institute of Agriculture and Life Science, Seoul National University, Seoul 08826, Korea; ykeys2584@snu.ac.kr; 5 Department of Animal Science, University of California, Davis, CA 95616, USA; yahliu@ucdavis.edu; 6 Pathway Intermediates, Seoul 06253, Korea; lhb@easybio.co.kr; 7 Department of Animal Resources Science, Dankook University, Cheonan 31116, Korea

**Keywords:** diarrhea, growth performance, gut microbiota, ileal gene expression, immune responses, intestinal morphology, nutrient digestibility, weaned pigs, yeast cell wall

## Abstract

**Simple Summary:**

Post-weaning stress can substantially affect performance of weaned pigs as well as overall pig production, and thus, a practical approach is needed to improve their performance by alleviating the stress that can cause intestinal barrier dysfunction of weaned pigs. There are potential ways to solve the concern in swine production, but dietary yeast cell wall in weaner diets may be one possible solution. The results of the present study suggest that dietary yeast cell wall improves growth performance of weaned pigs by enhancing gut health and provide its potential mechanism.

**Abstract:**

Dietary yeast cell wall products (YCW) are recognized as a feed additive due to multifunctional benefits by the biological response modulators. Thus, this study was conducted to verify a potential advantage of YCW for improving growth performance, nutrient digestibility, immune responses, and intestinal health and microbiota of weaned pigs. A total of 112 weaned pigs (7.99 ± 1.10 kg of body weight; 28 days old) were arbitrarily allocated to two experimental treatments with eight pigs (four barrows and four gilts) per pen and seven replicate pens per treatment in a completely randomized block design (block = BW and sex): (1) a basal diet based on corn and soybean meal (CON) and (2) CON + 0.05% YCW. The experimental period was for 4 weeks. There were no differences in final body weight, average daily feed intake, and gain-to-feed ratio between dietary treatments. In contrast, pigs fed YCW had higher average daily gain (*p* = 0.088) and apparent ileal digestibility of DM (*p* < 0.05) and energy (*p* = 0.052) and lower diarrhea frequency (*p* = 0.083) than those fed control diet (CON). Pigs fed YCW also had a higher (*p* < 0.05) ratio between villus height and crypt depth, villus width and area, and goblet cell counts in the duodenum and/or jejunum than those fed CON. Dietary YCW decreased (*p* < 0.05) serum TNF-α and IL–1β of weaned pigs on day 7 and 14, respectively, compared with CON. Furthermore, pigs fed YCW had higher (*p* < 0.05) ileal gene expression of claudin family, occludin, MUC1, INF-γ, and IL-6 and lower (*p* < 0.05) that of TNF-α than those fed CON. Lastly, there were no differences in the relative abundance of bacteria at the phylum level between CON and YCW. However, dietary YCW increased (*p* < 0.05) the relative abundance of genera *Prevotella* and *Roseburia* compared with CON. This study provided that dietary YCW improved growth rate, nutritional digestibility, and intestinal health and modified immune responses and intestinal microbiota of weaned pigs.

## 1. Introduction

Because of the potential effects of environmental stressors, the weaning period is a life stage that can substantially affect pig production. In this important period, weaned pigs must rapidly adapt to nutritional, immunological, and psychological changes [1]. Indeed, piglets can suffer from symptoms such as reduced feed intake, diarrhea, and villous atrophy of the small intestine that negatively affect digestion and absorption of nutrients during the early weaning stage [2]. In addition, weaning stress can induce changes in the intestinal structure and function as well as intestinal microbiota of piglets. Moreover, intestinal barrier function disorders caused by weaning stress can disrupt the intestinal microecological balance of piglets, potentially leading to the invasion of pathogens in the intestine and various disorders such as inflammation and diarrhea [3].

Including feed additives in the diet of piglets is a practical approach to alleviating intestinal barrier dysfunction during the weaning period [4]. Yeast or yeast-based products have been reported beneficial prebiotic effects on growth performance, intestinal morphology, and immunity of pigs, which can preventing various diseases [4,5]. Yeast cell wall (YCW) is thought to be effective due to its components, which consists of specific sugar types such as mannan oligosaccharides, α-D-mannans, and β-D-glucans [6]. Previous studies have reported that yeast-based products may be an effective alternative to antibiotics as without differences in growth performance, nutrient digestibility, and intestinal morphology in piglets [7,8]. Indeed, the addition of YCW or YCW-based products in weaned pig diets has been shown to increase villus heights and villus-to-crypt ratio [7,9]. In addition, prebiotics derived from YCW can boost the production of immunoglobulins and antibodies, which play a crucial role in the immunity of weaned pigs [10,11]. However, the physiological correlation between dietary YCW as prebiotic effects and pig’s gut health in a weaning stress condition needs to be further supported. The objective of this study, therefore, was to investigate the effects of YCW on health of weaned pigs, focusing on their growth performance, nutrient digestibility, immune responses, and intestinal health and microbiota.

## 2. Materials and Methods

### 2.1. Experimental Design and Sample Collection

All animal protocols for this experiment were approved by the Institutional Animal Care and Use Committee of Chungnam National University, Daejeon, Korea (approval #201909A-CNU-163). In total, 112 weaned pigs (Duroc × Landrace × Yorkshire) were arbitrarily allocated to two experimental treatments with eight pigs (4 barrows and 4 gilts) per pen and seven replicate pens per treatment in a completely randomized block design (block = BW and sex). The diets in the present experiment were (1) a basal diet based on corn and soybean meal (CON) and (2) CON + 0.05% dietary YCW. Pigs were housed in the same sized pen (2 m × 2 m) with a slatted plastic floor throughout the study. Environmental conditions are controlled by an automatic system that maintains the ambient temperature at 25–28 °C, and the lighting program is adjusted in a 12 h light/dark cycle. The average initial BW of the pigs was 7.99 ± 1.10 kg. At the end of the experiment, one randomly selected pig in each pen was anesthetized via an intramuscular injection of 2 mL succicholine (Ilsung Pharm. Co. Ltd., Seoul, Korea). Immediately after anesthesia, these pigs were sacrificed by CO_2_ gas for collecting ileal digesta samples, and the samples were stored at −20 °C until analysis. For histological analysis, a section (3 cm in length) of the middle duodenum, jejunum, and ileum were washed and fixed in 10% formalin [12]. Other pieces of distal ileum (5 cm in length) were used to collect ileum mucosa, which was fixed on glass slides and stored at −80 °C until analysis.

### 2.2. Diets

The formula of CON diet met or exceeded the nutritional requirements of weaned pigs based on NRC [13] (Table 1). The YCW product was obtained from a commercial supplier (CP: 29.58%; crude fiber: 3.69%; crude ash: 5.88%; beta-glucan 28.20%; Pathway Intermediates, Seoul, Korea). The pigs had free access to the respective diets and water for 28 days.

### 2.3. Growth Performance

Pig BW and feed disappearance were recorded individually on day 1, 7, 14, 21, and 28 after weaning to determine average daily gain (ADG), average daily feed intake (ADFI), and ratio between gain and feed intake (G:F) for growth performance. Each pig was monitored daily for diarrhea during the first 2 weeks after weaning; observations by two independent evaluators were quantified with a score on a scale of 1 to 5: 1 = solid excreta, 2 = damp excreta, 3 = shapeless excreta, 4 = semi-liquid excreta, and 5 = liquid severe excreta. Diarrhea frequency was calculated by counting pen days in which the average diarrhea score of individual pigs in each pen was ≥4.

### 2.4. Nutrient Digestibility

During the last week, pigs were fed with 0.2% Cr_2_O_3_ as a non-digestible indicator. Fecal samples from one randomly selected pig in each pen were obtained by rectal palpation for 3 days after an initial 4 days adjustment period and were stored at −20 °C until analysis. Diets, ileal digesta, and excreta samples were dried and pulverized by cyclone mill (Foss Tecator Sycltec 1093; Hillerød, Denmark) and were determined for DM, CP, and energy by bomb calorimeter (Parr 1281 Bomb Calorimeter; Parr Instrument Co., Moline, IL, USA) [14]. Concentration of chromium in samples was detected by an absorption spectrophotometer (Hitachi Z 5000 Absorption Spectrophotometer, Hitach High-Technologies Co., Tokyo, Japan) to determine the nutrient digestibility of weaned pigs according to previous study [15]. The apparent ileal digestibility (AID) and apparent total tract digestibility (ATTD) of DM, CP, and energy of each diet were determined as reported previously [16].

### 2.5. Intestinal Morphology

The intestinal morphology were measured as previously reported [12]. Briefly, fixed intestinal tissue samples were prepared by staining with hematoxylin and eosin, and their images were obtained by fluorescence microscopy (TE2000; Nikon, Tokyo, Japan). These images were used to analyze intestinal morphology such as villus (height, width, and area), crypt depth, ratio between villus height and crypt depth (VH:CD), and goblet cell counts. The means of each intestinal morphology measure (listed above) were used for analysis.

### 2.6. Immune Responses

On day 0, 7, and 14 after weaning, whole blood samples of one randomly selected pig in each pen were collected by vacuum tubes with EDTA (Becton Dickinson Vacutainer Systems, Franklin Lakes, NJ, USA), and the number of white blood cells (WBC) were analyzed by a hematology analyzer (scil Vet abc hematology analyzer; scil animal care company, F-67120 Altorf, France). Using serum samples on day 0, 7, and 14 after weaning, the porcine ELISA kit (R&D Systems Inc., Minneapolis, MN, USA) was used to measure the immune responses such as cortisol, tumor necrosis factor-α (TNF-α), transforming growth factor-β1 (TGF-β1), interleukin-1β (IL-1β), and interleukin-6 (IL-6). All cytokine measurements from serum samples were obtained according to a previously reported method [17]. The intra-assay coefficients of variation for cortisol, TNF-α, TGF-β1, IL-1 β, and IL-6 were 9.2, 6.9, 2.9, 7.2, and 5.1%, respectively, while the respective inter-assay coefficients of variation were 21.2, 9.2, 9.1, 8.7, and 7.4%.

### 2.7. Gene Expression Profiles in Ileum

Total RNA was collected from ileum mucosal samples using a HiGeneTM Total RNA Prep Kit (Biofact; Daejeon, Korea). Each sample contained two technical and three biological replicates (seven samples per treatment); the concentration and quality of the RNAs were checked using NanoDrop ND-1000 (NanoDrop Technologies; Wilmington, DE, USA). Subsequently, cDNAs were synthesized by using a Quantitect Reverse Transcription Kit (Qiagen, GmbH; Hilden, Germany). Quantitative real-time polymerase chain reaction (qRT-PCR) was performed using a StepOnePlus Real-Time PCR system (Applied Biosystems; Foster City, CA, USA), SFCgreenI (Biofact) and gene-specific designed primers (Bioneer; Daejeon, Korea). The primer sequences were designed using Primer Express Software v3.0.1 (Applied Biosystems) or by reference to previous studies (Table 2) [18]. The relative quantification of gene expression was determined using the 2^−ΔΔCt^ method, and 18S rRNA was used as an internal control for quantification of target genes.

### 2.8. 16s rRNA Sequencing for Fecal Microbiota

Fecal samples from three selected pigs per dietary treatment for verifying microbiota changes were obtained on the last day (35 days after weaning). Genomic DNA was collected from excreta samples by QIAamp DNA Stool Mini Kit (Qiagen, Hilden, Germany) based on their protocol. The concentration and quality of genomic DNA were checked using NanoDrop ND-1000 spectrophotometer (NanoDrop Technologies, DE, USA). Genomic DNA was stored for analysis at −20 °C. The V3–V4 region of the 16S rRNA gene was amplified by the PCR step using featured primers as listed previously [19]. The amplicons were sequenced by the Illumina MiSeq platform based on the directions of the manufacturer. All sequencing was conducted at Macrogen Inc. (Seoul, Korea). Raw sequence data were processed by the Mothur software, and low-quality sequences were eliminated [20]. Sequencing errors and chimeras were eliminated using the standard operating procedures for 454 sequences during Mothur processing [19]. The remaining high-quality sequences were categorized into operational taxonomic units (OTUs) clustering according to an identity cutoff of 97% [21]. The sequence number was normalized by randomly subsampling for downstream analyses of microbial alpha diversity such as phylogenetic information, observed OTUs, Chao1, Shannon, and Simpson index and beta diversity (PCoA; principal coordinates analysis).

### 2.9. Statistical Analyses

Results were analyzed using the GLM procedure in SAS (SAS Inst. Inc., Cary, NC, USA) with “pen” set as the experimental unit. The statistical model for growth performance, AID and ATTD, intestinal morphology, and immune responses of weaned pigs included effects of dietary treatment as a main effect and blocks as covariates. An χ^2^ test was used to analyze the diarrhea frequency. Normality of data was verified, and outliers were identified for all data. However, no outliers were detected and removed from the dataset. Results are presented as mean ± SEM. Statistical significance and tendency were set at *p* < 0.05 and 0.05 ≤ *p* < 0.10, respectively. The relative quantification of gene expression for tight junction proteins and cytokines was performed using Prism 5.0 software (GraphPad Software; La Jolla, CA, USA), and alpha diversity data were showed as mean ± standard deviation. All associated gene expression and microbiota data were analyzed by *t*-test with *p* < 0.05 considered as statistical significance.

## 3. Results

### 3.1. Growth Performance

Supplementation of YCW in the weaning diet tended to increase (*p* < 0.10) the ADG of weaned pigs over the entire experimental period compared with the CON (Table 3). In contrast, over this period, there were no differences in final BW, ADFI, and G:F between dietary treatments. However, pigs fed the YCW diet tended to have a lower (*p* < 0.10) diarrhea frequency than those fed a CON diet for the first 2 weeks after weaning (Table 3).

### 3.2. Nutrient Digestibility and Intestinal Morphology

The AID and ATTD of DM, CP, and energy of weaned pigs are shown in Table 4. Pigs fed the YCW diet had greater (*p* < 0.05) AID of DM and tended to have greater (*p* < 0.10) AID and ATTD of energy than those fed the CON diet. As shown in Table 4, dietary YCW significantly increased (*p* < 0.05) VH:CD, villus width, and villus area in the duodenum compared with CON. However, YCW supplementation in the weaning diet did not affect villus height or crypt depth of the duodenum. Pigs fed the YCW diet tended to have a higher (*p* < 0.10) VH:CD in the jejunum and higher (*p* < 0.10) villus width and villus area in the ileum than those fed the CON diet. Moreover, the YCW group showed a significantly increased (*p* < 0.05) number of goblet cells in the duodenum and jejunum compared with the CON group, although there was no difference in the number of goblet cells in the ileum.

### 3.3. Immune Responses and Gene Expression of Tight Junction Proteins

**Figure 1:**
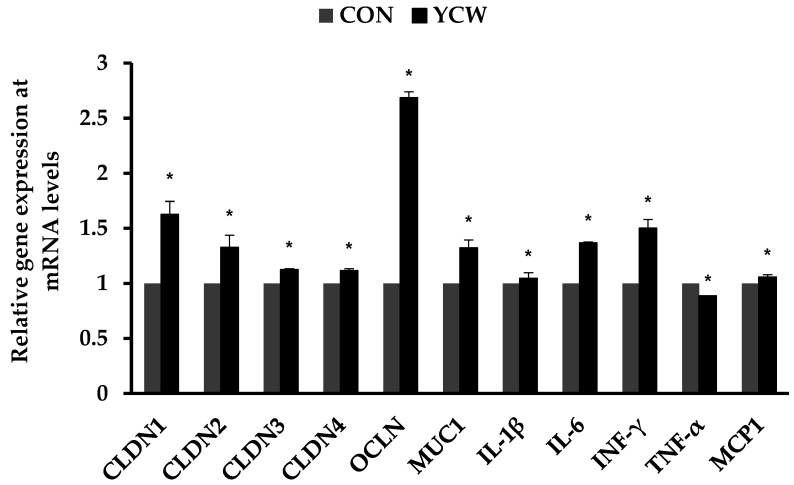
Relative gene expression levels of tight junction proteins and inflammatory cytokines in the ileal tissue of weaned pigs. n = 7. CON = a basal diet; YCW = CON with 0.05% dietary yeast cell wall; CLDN1 = claudin-1; CLDN2 = claudin-2; CLDN3 = claudin-3; CLDN4 = claudin-4; OCLN = occludin; MUC1 = mucin 1; IL-1β = interleukin-1β; IL-6 = interleukin-6; INF-γ = interferon-γ; TNF-α = tumor necrosis factor-α; MCP1 = monocyte chemoattractant protein 1. Data were analyzed by t-tests. * Different between CON and YCW (*p* < 0.05).

As shown in Table 5, dietary YCW tended to reduce (*p* < 0.10) the number of WBC on day 14 compared with CON. Interestingly, pigs fed the YCW diet had significantly lower (*p* < 0.05) serum TNF-α on day 7 and IL-1β on day 14, respectively, compared with those fed the CON diet. Additionally, the YCW group had higher (*p* = 0.05) serum TGF-β1 on day 14 than the CON group. However, the pigs fed the YCW diet apparently had no effect on the concentration of serum cortisol or IL-6. In addition, dietary YCW increased (*p* < 0.05) gene expression of the claudin family, occludin, mucin-1, IL-1β, IL-6, INF-γ, and monocyte chemoattractant protein-1 in the ileum compared with CON (Figure 1). In contrast, the YCW group decreased (*p* < 0.05) gene expression of TNF-α in the ileum compared with the CON group.

### 3.4. Diversity of Intestinal Microbiota

**Figure 2:**
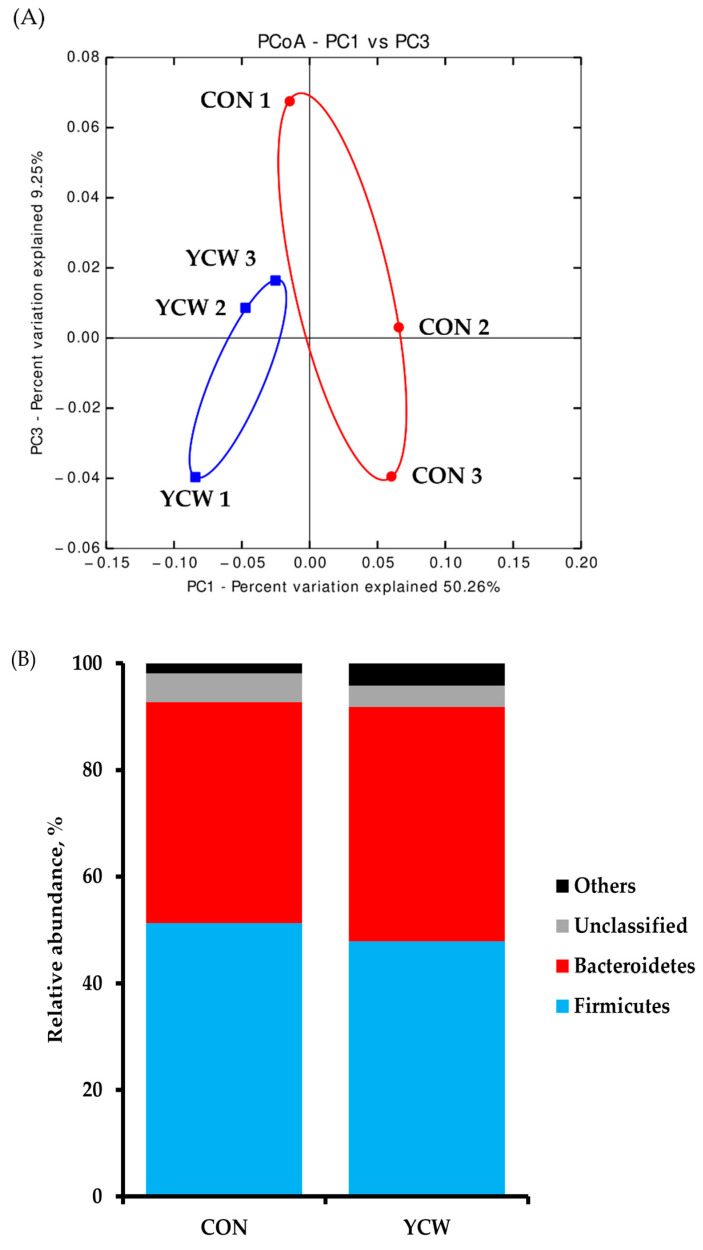

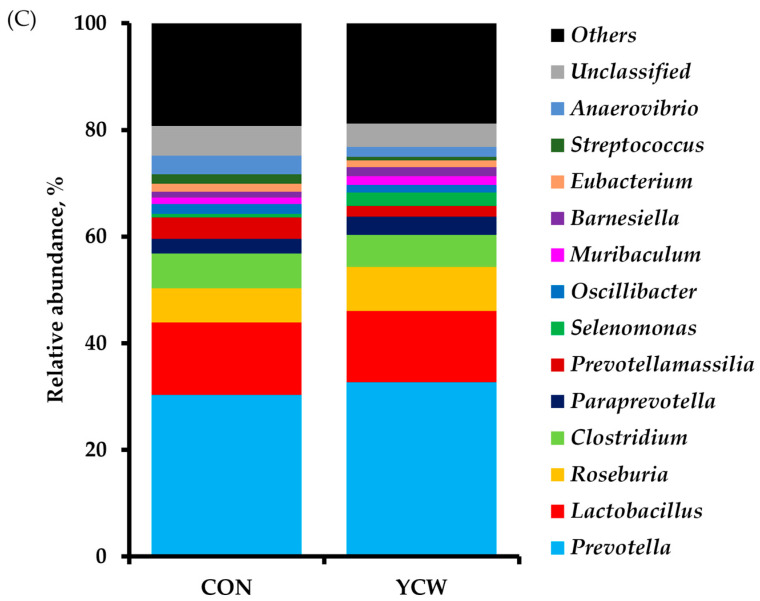
Principal coordinate analysis (PCoA) plot for weighted UniFrac distances in gut microbial communities from piglets’ feces of each dietary treatment (**A**). Taxonomic composition shows relative abundance of fecal microbiota in each treatment group at phylum level (**B**) and genus (**C**) level. n = 3. CON = a basal nursery diet (red, ○); YCW = CON with 0.05% dietary yeast cell wall (blue, □).

The result of diversity in the fecal bacterial community is shown in Table 6. The total sequence numbers were 128,647 ± 759 reads in the CON group and 122,242 ± 44,506 reads in the YCW group. The differences in alpha diversity indexes obtained between dietary treatments were not significant in the Chao1, Shannon, and Simpson diversity indices. The PCoA plot of the weighted UniFrac distances, such as beta diversity, illustrates the composition of microbial communities between the CON and YCW groups (Figure 2A). The microbial communities visually formed the distinct separation between CON and YCW groups on day 35. A comparison of the relative abundance of taxonomic groups at the phylum and genus levels between CON and YCW are shown in Figure 2. The relative abundance of bacteria at the phylum level were not significantly different between CON and YCW pigs (Figure 2B). However, weaned pigs fed YCW increased (*p* < 0.05) relative abundance of *Prevotella* and *Roseburia* compared with those fed CON (Figure 2C) at the genus level.

## 4. Discussion

In the present study, dietary YCW did not affect ADG, ADFI, and G:F of weaned pigs for the first 2 weeks after weaning. In addition, the diarrhea frequency was increased in the CON group for 2 weeks after weaning, which may be related to weaning events [2,3,22]. However, dietary YCW tended to increase ADG in weaned pigs when considered over the entire experimental period, suggesting that YCW supplementation may improve growth performance. Moreover, YCW supplementation reduced the diarrhea frequency of weaned pigs. These observations are in agreement with those of previous studies, in which the improved growth performance, nutrient absorption, and frequency of diarrhea are related to feed ingredients, intestinal morphology, and gut microbiota [4,5]. Furthermore, another study showed supplementation of yeast-based products increased the growth performance of weaned pigs [23]. Interestingly, the observations in the present study are consistent with those of a previous study, in which dietary yeast product supplementation did not affect ADG for the first 3 weeks after weaning but increased the ADG of weaned pigs following 6 weeks of consumption [24]. Therefore, we postulate that the ingestion of YCW over relatively long periods may have positive effects on the intestinal morphology and, thereby, increase nutrient digestibility and improve the growth performance of weaned pigs.

The AID of DM and ATTD of energy of weaned pigs were increased with YCW supplementation in the present study. Similarly, a previous study showed dietary YCW improved the growth performance and nutrient digestibility of weaned pigs [25]. Intestinal morphology, including villus height, width, and area, as well as crypt depth and the goblet cell counts, is generally associated with nutrient digestion and absorption [25,26], and it is known that yeast-based products improve nutrient digestibility due to their beneficial effects on intestinal morphology and function [25,26]. The cell wall of yeast is composed of mannoproteins, β-(1,3) or (1,6)-D-glucans, chitin, and glycophospholipid proteins related to the plasma membrane [8]. The YCW components include specific sugar types, such as β-D-glucans, α-D-mannans, and mannan oligosaccharides, which have prebiotic effects [8]. In the present study, villus width and area and VH:CD were increased in the ileum and duodenum, while the goblet cell counts increased in the duodenum and jejunum as a result of YCW supplementation: these results agree with previous observations, in which prebiotics derived from YCW increased the proliferation of intestinal cells and the concentration of glycoconjugates in the mucins [7,9].

The tight junction is a crucial structure related to the epithelial barrier function of the intestinal tract. The physical barrier function in the intestine is associated with the tight junction structure between intestinal epithelial cells. Therefore, the health of the intestinal tight junction is crucial to maintaining polarity in intestinal epithelial cells and preventing a material spill in the epithelial cell gap. Claudin and occludin members of the tetraspan family related to transmembrane proteins form part of the gut structure and play roles in the epithelial barrier function of the intestine [27]. Results from a previous experiment indicate that the gene expression of claudin and occludin proteins could be an indicator of epithelial barrier function in the intestine [27]. In the present experiment, we observed that dietary YCW supplementation significantly increased the gene expression of claudin, occludin, and mucin proteins in the ileum of weaned pigs. This is consistent with the results of previous studies, which showed that the supplementation of yeast-based products upregulated the gene expression of tight junction proteins, including claudin and occludin in the intestinal mucosa [9] and improved barrier function via the increased thickness of the mucosal layer and number of mucosal macrophages [28].

We performed 16S rRNA gene analysis to evaluate the effects of YCW on fecal microbiota of weaned pigs. During weaning period, an alteration of dynamic microenvironment occurs in the piglet’s intestinal tract with the changes in diets and age [29]. In the present study, YCW supplementation increased genera *Prevotella* and *Roseburia*. This is consistent with the previous studies that yeast and its derivatives, as probiotic effects, can modify the community of gut microbiota and reduce diarrhea, which improve the health of early-weaned piglets [30]. *Prevotella* strains are the most dominant bacterial community at genus level [31]. In addition, *Prevotella* and *Roseburia* strains are related to the production of short chain fatty acids (SCFAs) by the fermentation of indigestible polysaccharides in the intestine [32]. These *Prevotella* and *Roseburia* strains promote nutrient digestibility; thus, YCW supplementation increases the nutrient digestibility of weaned pigs possibly through the intestinal microbiota, especially *Prevotella* and *Roseburia*. It is known that the SCFAs, such as acetate, propionate, and butyrate, regulate the barrier function and immune system and prevent the establishment of pathogenic communities in the gut [32]. Therefore, the observation of microbiota in the present study supports that YCW supplementation could improve the nutrient digestibility with the enhancement of epithelial barrier and immune function in weaned pigs by increasing the relative abundance of *Prevotella* and *Roseburia*.

It has been reported that stress factors during the weaning period induce inflammation in the intestine and serum of weaned pigs, which leads to an increase in typical pro-inflammatory cytokines, such as TNF-α, IFN-γ, IL-β1, IL-6, and TGF-β1 [3]. In addition, both pro- and anti-inflammatory cytokines play a key role in the regulation of immune responses and intestinal barrier function [33]. In the present study, YCW supplementation increased the gene expression of some pro-inflammatory cytokines, such as IL-β1 and IL-6, and reduced that of TNF-α; furthermore, the gene expression of anti-inflammatory cytokine, INF-γ, was upregulated in the ileum. Moreover, it was observed that serum TNF-α and IL-β1 concentrations were decreased on day 7 and 14, respectively, whereas serum TGF-β1 concentration was increased on day 14 in weaned pigs fed YCW. The TGF-β1 considered as an indicator of anti-inflammatory cytokines in the present study generally functions as both an immune-suppressor and -enhancer that modulate the proliferation and differentiation of T and B cells. On the other hand, the TNF-α and IL-6, which are typical pro-inflammatory cytokines, stimulate macrophages to induce inflammatory responses and regulate immune responses [34]. These results suggest that YCW supplementation in weaning diets is beneficial for immune-modulation. Previous studies are in agreement with the concern [10,11]: YCW products stimulated the immune system to increase antibody production because the β-D-glucans from YCW bind to receptors causing cytokine cascades and improved macrophage function [35]. A previous study demonstrated that the α-D-mannans in YCW bind to mannose-specific receptors and, thereby, prevent the adhesion of pathogens to the intestinal barrier [10]. Therefore, the present study indicates that the dietary YCW supplementation could attenuate the inflammation caused by weaning stress.

## 5. Conclusions

In summary, supplementation with dietary YCW in weaning diets for a long period (28 days) resulted in a significant improvement of nutrient digestibility and intestinal health and modified the intestinal microbiota of weaned pigs. These results provide fundamental evidence to support the future development of the dietary YCW as a functional feed additive for weaned pigs.

## Figures and Tables

**Table 1:** Ingredient composition of basal diet for weaned pigs (as-fed basis).

Items	Basal Diet
**Ingredient, %**	
Corn	53.90
Soybean meal (44%)	15.00
Soy protein concentrate	7.50
Whey powder	12.50
Soybean oil	2.30
Spray dried porcine plasma	2.50
Fish meal, combined	3.00
Limestone	1.20
Monocalcium phosphate	0.80
Vitamin-mineral premix ^1^	0.40
L-Lysine-HCl	0.35
DL-Methionine	0.15
L-Threonine	0.10
Zinc oxide	0.30
Total	100.00
**Calculated energy and nutrient contents**	
Metabolizable energy, MJ/kg	14.24
Crude protein, %	21.69
Calcium, %	0.89
Phosphorus, %	0.68
Lysine, %	1.55

^1^ per kg of diet: 12,000 IU vitamin A, 2500 IU vitamin D_3_, 30 IU vitamin E, 3 mg vitamin K_3_, 15 mg _D_-pantothenic acid, 40 mg nicotinic acid, 400 mg choline, 12 μg vitamin B_12_, 90 mg Fe from iron sulfate; 8.8 mg Cu from copper sulfate; 100 mg Zn from zinc oxide; 54 mg Mn from manganese oxide; 0.35 mg I from potassium iodide; 0.30 mg Se from sodium selenite.

**Table 2:** Gene-specific primer sequences of tight junction protein and inflammatory cytokine genes in the ileal tissue.

Items ^1^	Forward (5′-3′)	Reverse (5′-3′)
CLDN1	AGAAGATGCGGATGGCTGTC	CCCAGAAGGCAGAGAGAAGC
CLDN2	TCCTCCCTGTTCTCCCTGATAG	CCTTGCAGTGGGCAGGAA
CLDN3	GATGCAGTGCAAAGTGTACGA	GTCCTGCACGCAGTTGGT
CLDN4	TATCATCCTGGCCGTGCTA	CATCATCCACGCAGTTGGT
OCLN	GGAGTGATTCGGATTCTGTCTATGCT	CGCCTGGGCTGTTGGGTTGA
MUC1	CCCTGGCCATCATCTATGTC	TGCCCACAGTTCTTTCGTC
IL-1β	GCCCTGTACCCCAACTGGTA	CCCAGGAAGACGGGCTTT
IL-6	GCGCAGCCTTGAGGATTTC	CCCAGCTACATTATCCGAATGG
INF-γ	GAGCCAAATTGTCTCCTTCTAC	CGAAGTCATTCAGTTTCCCAG
TNF-α	CTTGGGTTTGGATTCCTGGAT	CTTCCCTGGCAGCCACAT
MCP1	TCCCACACCGAAGCTTGAAT	CACAGGAGGGCTGCAGAGA
18S rRNA	GGCTACCACATCCAAGGAAG	TCCAATGGATCCTCGCGGAA

^1^ CLDN1 = claudin-1; CLND2 = claudin-2; CLDN3 = claudin-3; CLDN4 = claudin-4; OCLN = occludin; MUC1 = mucin-1; IL-1β = interleukin-1β; IL-6 = interleukin-6; INF-γ = interferon-γ; TNF-α = tumor necrosis factor-α; MCP1 = monocyte chemoattractant protein-1.

**Table 3:** Growth performance of weaned pigs fed diets with or without dietary yeast cell wall.

Item ^1^	CON	YCW	SEM	*p*-Value
**Day 1 to 7**				
Initial BW, kg	7.99	7.98	0.43	0.995
Final BW, kg	9.21	9.33	0.40	0.842
ADG, g/d	203.93	223.69	15.81	0.394
ADFI, g/d	258.44	278.57	13.93	0.327
G:F, g/g	0.78	0.80	0.03	0.710
Diarrhea ^2^, %	6.12	0.00		0.243
**Day 8 to 14**				
Initial BW, kg	9.21	9.33	0.40	0.842
Final BW, kg	12.60	12.94	0.45	0.598
ADG, g/d	376.19	401.59	11.96	0.159
ADFI, g/d	512.31	545.45	14.10	0.123
G:F, g/g	0.73	0.74	0.01	0.882
Diarrhea ^2^, %	18.37	10.20		0.317
**Day 1 to 14**				
Initial BW, kg	7.99	7.98	0.43	0.995
Final BW, kg	12.60	12.94	0.45	0.598
ADG, g/d	307.29	330.43	11.68	0.186
ADFI, g/d	410.76	438.70	11.82	0.121
G:F, g/g	0.75	0.75	0.01	0.783
Diarrhea ^2^, %	12.24	5.10		0.083
**Day 15 to 28**				
Initial BW, kg	12.60	12.94	0.45	0.598
Final BW, kg	19.77	20.53	0.65	0.422
ADG, g/d	512.50	542.35	16.83	0.234
ADFI, g/d	856.03	863.88	30.49	0.859
G:F, g/g	0.60	0.63	0.01	0.118
**Day 1 to 28**				
Initial BW, kg	7.99	7.98	0.43	0.995
Final BW, kg	19.77	20.53	0.65	0.422
ADG, g/d	420.87	448.19	10.41	0.088
ADFI, g/d	648.07	666.96	18.36	0.481
G:F, g/g	0.65	0.67	0.01	0.101

^1^ CON = a basal diet; YCW = CON with 0.05% dietary yeast cell wall; ADG = average daily gain; ADFI = average daily feed intake; G:F = ratio between gain and feed intake; n = 7. ^2^ Diarrhea = (number of pigs with diarrhea/number of pen days) × 100.

**Table 4:** Nutrient digestibility and intestinal morphology of weaned pigs fed diets with or without dietary yeast cell wall.

Item ^1^	CON	YCW	SEM	*p*-Value
**Apparent ileal digestibility, %**				
Dry matter	79.76	80.75	0.27	0.024
Crude protein	72.27	71.68	2.56	0.873
Energy	75.77	78.14	0.78	0.052
**Apparent total tract digestibility, %**				
Dry matter	84.06	86.02	0.90	0.147
Crude protein	76.17	76.11	1.02	0.972
Energy	81.61	83.46	0.69	0.085
**Duodenum**				
Villus height, μm	317.73	356.68	16.12	0.113
Crypt depth, μm	266.48	236.14	12.31	0.107
VH:CD, μm/μm	1.19	1.52	0.05	0.001
Villus width, μm	137.83	162.61	6.76	0.024
Villus area, μm^2^	38,286	46,226	1688	0.006
Goblet cell, n	9.93	13.95	1.10	0.024
**Jejunum**				
Villus height, μm	307.05	338.81	19.42	0.349
Crypt depth, μm	263.97	238.44	19.85	0.381
VH:CD, μm/μm	1.19	1.42	0.08	0.077
Villus width, μm	159.44	173.99	8.58	0.254
Villus area, μm^2^	40,550	42,568	1491	0.357
Goblet cell, n	9.03	13.27	1.05	0.014
**Ileum**				
Villus height, μm	393.54	416.01	10.95	0.172
Crypt depth, μm	256.93	242.39	13.98	0.476
VH:CD, μm/μm	1.55	1.74	0.09	0.153
Villus width, μm	143.70	173.31	10.08	0.060
Villus area, μm^2^	32,201	41,849	3691	0.089
Goblet cell, n	14.71	14.94	0.86	0.851

^1^ CON = a basal diet; YCW = CON with 0.05% dietary yeast cell wall; VH:CD = ratio between villus height to crypt depth; n = 7.

**Table 5:** Immune responses of weaned pigs fed diets with or without dietary yeast cell wall.

Item ^1^	CON	YCW	SEM	*p*-Value
**White blood cell, ×10^3^/μL**				
Day 0	12.51	10.43	1.51	0.348
Day 7	14.90	15.31	0.93	0.757
Day 14	21.81	16.95	1.79	0.081
**Cortisol, ng/mL**				
Day 0	44.73	45.40	2.03	0.821
Day 7	48.23	46.87	1.00	0.363
Day 14	50.45	49.86	1.74	0.467
**Tumor necrosis factor-α, pg/mL**				
Day 0	65.84	64.12	5.65	0.836
Day 7	126.57	81.91	13.48	0.039
Day 14	119.43	111.88	13.63	0.702
**Transforming growth factor-β1, pg/mL**				
Day 0	1397.69	1310.72	106.45	0.576
Day 7	1248.89	1200.29	95.41	0.726
Day 14	1129.77	1474.58	107.88	0.050
**Interleukin-1β, pg/mL**				
Day 0	28.97	25.73	4.69	0.635
Day 7	39.29	41.06	2.10	0.564
Day 14	38.88	27.20	2.49	0.008
**Interleukin-6, pg/mL**				
Day 0	139.41	140.44	2.90	0.808
Day 7	147.84	143.56	3.62	0.420
Day 14	143.12	138.80	3.90	0.448

^1^ CON = a basal diet; YCW = CON with 0.05% dietary yeast cell wall; n = 7.

**Table 6:** Alpha diversity analysis for fecal microbiota of weaned pigs fed diets with or without dietary yeast cell wall.

Item ^1^	CON	YCW	*p*-Value
Number of read sequences	128,647 ± 759	122,242 ± 44,506	0.535
OTUs	286.00 ± 48.82	263.30 ± 31.09	0.432
Chao1	315.80 ± 51.97	285.10 ± 31.59	0.445
Shannon	5.33 ± 0.54	5.62 ± 0.26	0.193
Inverse Simpson	0.93 ± 0.03	0.96 ± 0.01	0.826

^1^ CON = a basal diet; YCW = CON with 0.05% dietary yeast cell wall; OTUs = operational taxonomic units; Chao1 = estimation of richness for OTUs definition; Shannon = explanation the number and evenness of species; inverse Simpson = probability of two randomly selected individuals in their habitat belong to the same species; n = 3.

## Data Availability

The data presented in this study are available from the corresponding author on request.
